# Assessment of the ability of the CHA_2_DS_2_-VASc scoring system to grade left atrial function by 2D speckle-tracking echocardiography

**DOI:** 10.1186/s12872-021-01908-8

**Published:** 2021-02-16

**Authors:** Marjan Hadadi, Reza Mohseni-Badalabadi, Ali Hosseinsabet

**Affiliations:** Cardiology Department, Tehran Heart Center, Tehran University of Medical Sciences, Karegar Shomali Street, Tehran, Islamic Republic of Iran

**Keywords:** Left atrium, Coronary artery disease, Two-dimensional speckle-tracking echocardiography, CHA_2_DS_2_-VASc score

## Abstract

**Background:**

The CHA_2_DS_2_-VASc scoring system is correlated with left atrial (LA) reservoir function in patients with atrial fibrillation (AF) rhythm or paroxysmal AF. We assessed the ability of CHA_2_DS_2_-VASc to grade LA function in patients with sinus rhythm who were candidates for coronary artery bypass grafting (CABG).

**Methods:**

This cross-sectional study recruited 340 consecutive candidates for CABG and categorized them according to their CHA_2_DS_2_-VASc scores as mild-, moderate-, and high-risk score groups with 34 (10%), 83 (24%), and 223 (66%) patients, respectively. LA function was evaluated via 2D speckle-tracking echocardiography in terms of global longitudinal strain and strain rate during the reservoir, conduit, and contraction phases. In-hospital mortality, postoperative AF, prolonged intensive care unit (ICU) stay, and prolonged mechanical ventilation were assessed.

**Results:**

LA strain and strain rate during the reservoir phase was statistically significantly lower in the high-risk score group than the low- and moderate-risk score groups (27.8 ± 6.9% vs 31.0 ± 5.0% vs 29.8 ± 6.1%, respectively; *P* = 0.004 and 2.6 ± 0.7 s^−1^ vs 2.9 ± 0.6 s^−1^ vs 2.9 ± 0.6 s^−1^, correspondingly; *P* = 0.009) and regarding LA strain and strain rate during the conduit phase (9.7 [7.1–12.5]% vs 12.9 [9.4–15.1]% vs 11.5 [9.1–13.8]%, correspondingly; *P* < 0.001 and 2.1 [1.6–2.7] s^−1^ vs 2.8 [2.4–3.6] s^−1^ vs 2.6 [2.2–3.0] s^−1^, respectively; *P* < 0.001). In addition, LA strain rate during the conduit phase was lower in the moderate-risk score group than the low-risk score group. After adjustments for possible confounders, these differences remained statistically significant. The risk of postoperative AF and prolonged ICU stay was highest in the high-risk score group (relative risk = 9.67 (1.31–71.43) and 8.05 (1.08–60.16), respectively; *P* = 0.026 and *P* = 0.042, respectively).

**Conclusions:**

LA reservoir and conduit functions decreased in the high-risk score group, which was accompanied by an increased risk of postoperative AF and prolonged ICU stay.

## Introduction

The CHA_2_DS_2_-VASc score is a scoring system that was first used for the risk assessment of cerebrovascular or thromboembolic events in patients with atrial fibrillation (AF). The factors evaluated are such clinical factors as congestive heart failure, left ventricular (LV) dysfunction (LV ejection fraction < 40%), hypertension, age, diabetes, stroke, transient ischemic attack, thromboembolism, sex (female), and vascular diseases including a prior myocardial infarction aortic plaque and peripheral arterial disease. This scoring system allocates 1 point to each factor except for stroke, transient ischemic attack, and thromboembolism (2 points for each), and age (2 points allocated to age ≥ 75 years and 1 point to 65 < age < 74 years) [1]. The application of this scoring system for the prediction of thromboembolic events is not restricted to patients with AF; it can also be applied to patients without AF such as those with heart failure [2] or chronic obstructive pulmonary disease [3] and those after coronary artery bypass grafting (CABG) [4, 5]. It can also predict the occurrence of AF in varieties of conditions such as diabetes [6], systemic lupus erythematosus [7], myocardial infarction [8], and cardiac surgeries including CABG [9–11]. In addition, the CHA_2_DS_2_-VASc score plays a role in the prediction of events other than stroke and AF such as failed reperfusion after thrombolytic therapy [12], the no-reflow phenomenon [13], and contrast-induced nephropathy [14].

Left atrial (LA) dysfunction in patients suffering from AF [15] or with a history of stroke [16] has been previously documented. The prognostic capabilities of LA function in predicting cardiac hospitalization and mortality [17], functional capacity [18], and paroxysmal AF [19] have also been demonstrated.

Two-dimensional speckle-tracking echocardiography (2DSTE) can assess deformations in LA myocardium and is deemed an accepted imaging modality for the evaluation of LA function [20]. Previous research has revealed that LA strain measured by 2DSTE is reduced in patients with AF rhythm or paroxysmal AF and high CHA_2_DS_2_-VASc or CHADS_2_ scores compared with those with low CHA_2_DS_2_-VASc or CHADS_2_ scores [21–23]. Nonetheless, whether or not the CHA_2_DS_2_-VASc scoring system is capable of identifying LA dysfunction in patients with sinus rhythm has yet to be determined. We sought to assess the ability of the CHA_2_DS_2_-VASc scoring system to grade LA function in a group of candidates for CABG who were in sinus rhythm.

## Materials and methods

### Study population

The present cross-sectional study recruited 340 candidates for CABG in a referral heart center between May 2019 and October 2019. The inclusion criterion was sinus rhythm, and the exclusion criteria were comprised of history of AF rhythm, history of cardiac surgery, pacemaker implantation, congenital heart diseases, cancer, inflammatory diseases, recent myocardial infarction (< 6 weeks, due to acute hemodynamic, inflammatory, and neuroendocrine changes), hypertrophic and restrictive cardiomyopathy, pericardial diseases, valvular heart diseases (moderate and more-than-moderate valvular regurgitation or any-degree valvular stenosis), left bundle branch block, poor echocardiography windows, thyroid disease, liver disease, and a creatinine level of more than 1.5 mg/dL. History taking and physical examinations were done after patient admission, and venous samples for the evaluation of the lipid profile, the fasting blood glucose level, and the cell blood count were drawn after overnight fasting on the morning of the first post-admission day. Diabetes was defined as a minimum fasting blood glucose level of 126 mg/dL in 2 separate samples or the consumption of insulin or oral antidiabetic agents. Hypertension was defined as a blood pressure of more than 140/90 mm Hg in 2 medical visits or the consumption of antihypertensive agents. In accordance with the latest guidelines of the American College of Cardiology/American Heart Association on the management of AF, the patients were assigned to 3 groups of low-risk score (CHA_2_DS_2_-VASc score = 0 for men and 1 for women), moderate-risk score (CHA_2_DS_2_-VASc score = 1 for men and 2 for women), and high-risk score (CHA_2_DS_2_-VASc scores ≥ 2 for men and ≥ 3 for women) with 34 (10%), 83 (24%), and 223 (66%) patients, respectively [24]. The clinical outcome was defined as in-hospital mortality, postoperative AF, prolonged stay in the intensive care unit (ICU), and prolonged mechanical ventilation time. A detailed explanation of our definition of postoperative AF and its registration process in our center has been previously provided [25]. Briefly, episodes lasting more than 30 s are considered postoperative AF. These episodes are diagnosed through continuous cardiac monitoring in the first 3 days after CABG and thereafter via electrocardiography if patients remain symptomatic until hospital discharge. A prolonged ICU stay is defined as a period of more than 8 days in the ICU (according to the mean value presented in the literature [26]), and a prolonged mechanical ventilation time is defined as mechanical ventilation exceeding 72 h. Twenty-eight (8%) patients refused CABG; consequently, the evaluation of the clinical outcome was done on 312 (92%) patients. The study population was divided into a low-risk score group (n = 30 [10%]), a moderate-risk score group (n = 79 [25%]), and a high-risk score group (n = 203 [65%]).

### Echocardiography

Standard echocardiography was performed in the left lateral decubitus position by a highly experienced cardiologist. One-lead electrocardiography monitoring was conducted during echocardiography. A commercial setting (Philips, Affinity 70C, Andover, MA, USA) with an S5-1 probe was used for image acquisition after patient admission. Linear LV diameters and LV wall thickness were measured in the parasternal long-axis view, and then LV mass index was calculated. LV end-diastolic and end-systolic volumes were measured in the apical 4- and 2-chamber views based on the biplane modified Simpson method, and then LV ejection fraction was calculated. With the aid of pulsed-wave Doppler, the mitral flow profile was depicted in the apical 4-chamber view, and early and late diastolic peak velocities (E and A, respectively) and the deceleration time of the E-wave were measured. Additionally, the systolic and diastolic peak velocities of the pulmonary vein (S and D, respectively) were measured. Systolic, early diastolic, and late diastolic myocardial peak velocities (s′, e′, and a′, correspondingly) were also measured by pulsed-wave tissue Doppler imaging in the medial and lateral mitral annuli in the apical 4-chamber view, and the average of the measured values was calculated. The Doppler-based measurements were repeated in 3 cardiac cycles, and their mean value was reported. All these measurements and calculations were done in keeping with the recommendations of the American Society of Echocardiography [27–29].

For 2DSTE, 3 consecutive cardiac cycles in the apical 4- and 2-chamber views were acquired in the expiration phase of the respiratory cycle by applying maximal effort for the exclusion of LA appendage or the orifice of the pulmonary vein. The images were acquired at an image rate of 47 ± 3 frames per second. The aCMQ option of QLAB software, version 12 (Philips, Andover, MA, USA) was utilized for the evaluation of the longitudinal systolic and diastolic deformations of LA myocardium in the 4- and 2-chamber views.

**Fig. 1:**
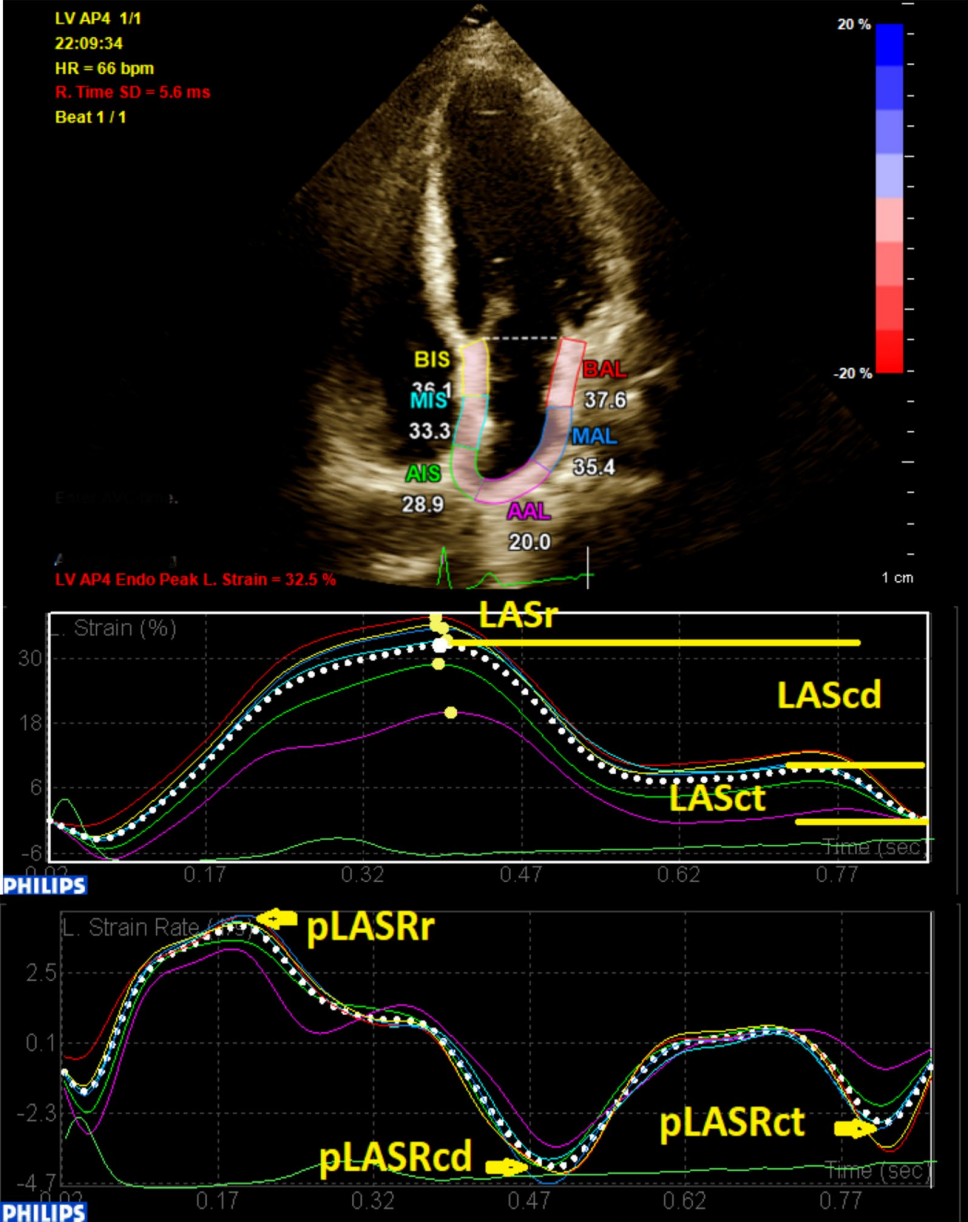
Two-dimensional speckle-tracking echocardiography for the evaluation of left atrial myocardial function is presented in the apical 4-chamber view. LAScd, Left atrial longitudinal strain during the conduit phase; LASct, Left atrial longitudinal strain during the conduit phase; LASr, Left atrial longitudinal strain during the reservoir phase; pLASRcd, Peak left atrial longitudinal strain rate during the conduit phase; pLASRct, Peak left atrial longitudinal strain rate during the contraction phase; pLASRr, Peak left atrial longitudinal strain rate during the reservoir phase

First, via the 3-point method, the center of LA roof and the medial and lateral mitral annuli at end-diastole were defined. Next, the endocardial and pericardial borders of LA myocardium were delineated with the software automatically, and the defined borders were manually adjusted to the true borders. Thereafter, the software illustrated the tracking of the mentioned borders during the cardiac cycle, and the operator ensured that the traced line tracked the true boundaries and made appropriate adjustments. If there were 2 and more segments with noisy signals after several attempts, the patient was excluded from the study. These steps were repeated for each stored cardiac cycle. The software set the 0 level at the initiation of the R-wave of electrocardiography. LA global longitudinal strain curve consisted of 3 components: a positive systolic peak, an early diastolic plateau, and a late diastolic trough. The difference between the peak and trough values was termed “LASr”, the difference between the plateau and peak values “LAScd”, and the difference between the trough and plateau values “LASct”. LA global longitudinal strain rate had 3 peaks: a positive systolic peak (pLASRr), a negative early diastolic peak (pLASRcd), and a negative late diastolic peak (pLASRct) (Fig. 1). These global deformation markers were measured in each view for 3 cardiac cycles, and their averaged values were reported. LASr and pLASRr are the markers of LA reservoir function, LAScd and pLASRcd are the indices of LA conduit function, and LASct and pLASRct are the parameters of LA contraction function. The measurements were done according to the recommendations of the American Society of Echocardiography [20].

The aCMQ option provided an LA volume-time curve, which enabled the operator to measure maximal, minimal, and pre-P LA volumes (LAVMx, LAVMi, and LAVp, respectively) for the calculation of the volumetric-derived parameters of LA function.

The parameters of LA reservoir function were as follows:

$${\text{total}}\,{\text{emptying}}\,{\text{fraction}}\, = \,100\, \times \,\left( {{\text{LAVMx}}\, - \,{\text{LAVM}}i} \right)/{\text{LAVMx}}$$

and

$${\text{expansion}}\,{\text{index}}\, = \,100\, \times \,\left( {{\text{LAVMx}}\, - \,{\text{LAVMi}}} \right)/{\text{LAVMi}}$$

The indices of LA conduit function were as follows:

$${\text{passive}}\,{\text{emptying}}\,{\text{fraction}}\, = \,100\, \times \,\left( {{\text{LAVMx}}\, - \,{\text{LAVp}}} \right)/{\text{LAVMx}}$$

and

$${\text{passive}}\,{\text{emptying}}\,{\text{percentage}}\,{\text{of}}\,{\text{total}}\,{\text{emptying}}\, = \,100\, \times \,\left( {{\text{LAVMx}}\, - \,{\text{LAVp}}} \right)/({\text{LAVMx}}\, - \,{\text{LAVMi}})$$

The parameters of LA contraction function were as follows:

$${\text{active}}\,{\text{emptying}}\,{\text{fraction}}\, = \,100\, \times \,\left( {{\text{LAVp}}\, - \,{\text{LAVMi}}} \right)/{\text{LAVp}}$$

and

$${\text{booster}}\,{\text{active}}\,{\text{emptying}}\,{\text{percentage}}\,{\text{of}}\,{\text{total}}\,{\text{emptying}}\, = \,100\, \times \,\left( {{\text{LAVp}}\, - \,{\text{LAVMi}}} \right)/({\text{LAVMx}}\, - \,{\text{LAVMi}})$$

2DSTE analyses were carried out after the completion of patient recruitment up to the end of January of 2020 by a cardiologist highly experienced in advanced echocardiography. The cardiologist was blind to the clinical data. Images of 51 (15%) patients were chosen randomly for the evaluation of intra- and interobserver variabilities. The second analysis was done 1 month after the completion of the first analysis, with the original operator and a second observer (another cardiologist highly experienced in advanced echocardiography) separately analyzing the images while blinded to the results of the first analysis.

### Statistical analysis

Continuous variables with normal distributions were summarized as mean ± SD and compared via the one-way analysis of variance. The post hoc analysis was done using the Dunnett T3 test; otherwise; variables were presented as the median and the interquartile range and compared via the Kruskal–Wallis H test. Additionally, the post hoc analysis was done using the adjusted Bonferroni test. Categorical data were presented as frequencies and percentages and compared via the χ^2^ test. If the expected count in 20% of the cells or more was less than 5, the Fisher exact test was employed. Generalized linear models were utilized the compare the longitudinal deformation markers of LA myocardium before and after adjustments for possible confounders. In the first step, variables with a *P* value of less than 0.15 were candidated for entrance in the adjusted analysis after the absence of collinearity was checked. In the second step, physiologically-related variables were selected according to published data. Confounders were considered to be systolic blood pressure, heart rate, hemoglobin levels, low-density lipoprotein levels, cigarette smoking, left circumflex artery stenosis, single-vessel disease, obesity, and nitrate use, all of which were entered in the adjusted analysis. The differences between the groups (parameter estimates) were presented as B and 95% Wald confidence intervals. Intra- and interobserver variabilities were evaluated with intraclass correlation coefficients and 95% levels of agreement. With respect to the clinical outcome, the incidence of events in the high-risk score group and the non–high-risk score groups (viz, low- and moderate-risk groups) was calculated so that the relative risk could be estimated with a 95% confidence interval. The statistical analyses were performed by applying IBM SPSS Statistics for Windows, version 24 (Armonk, NY: IBM Corp). A *P* value of less than 0.05 was regarded as statistically significant.

## Results

The study population was comprised of 340 patients, divided into a low-risk score group (n = 30 [10%]), a moderate-risk score group (n = 79 [25%]), and a high-risk score group (n = 203 [65%]). A risk score of 1 was reported in 4 (12%) patients in the low-risk score group and 66 (80%) in the moderate-risk score group; the remainder had a risk score of 2. In addition, in the high-risk score group, 90 (40%), 76 (34%), 34 (15%), 15 (7%), and 8 (4%) patients had risk scores of 2, 3, 4, 5, and 6, respectively.

### Clinical and laboratory data

The clinical and laboratory data of our study groups are demonstrated in Table 1. Regardless of the variables that constitute the CHA_2_DS_2_-VASc scoring system, the study groups were statistically different not only in terms of the consumption of insulin, oral antidiabetic agents, calcium-channel blockers, angiotensin-converting enzyme inhibitors or angiotensin II-receptor blockers, and diuretics but also in regard to left circumflex artery stenosis, single-vessel disease, and obesity.

**Table 1:** Clinical and laboratory data of the 3 groups of low-, moderate-, and high-risk CHA_2_DS_2_-VASc scores

Variable	Group			*P* value
	Low-Risk Score (N = 34)	Moderate-Risk Score (N = 83)	High-Risk Score (N = 223)	
**Clinical data**				
Age (y)	55 ± 6	59 ± 7^c^	64 ± 9^a,b^	< 0.001
Age < 65	34 (100)	68 (82)	113 (51)	< 0.001
65 ≤ Age < 75	0(0)	15 (18)	83 (37)	
Age ≥ 75	0 (0)	0(0)	27 (12)	
Female sex (%)	4 (12)	17 (21)	61 (27)	0.095
Body mass index (kg/m^2^)	27.9 ± 4.5	28.3 ± 4.6	27.4 ± 4.0	0.289
Body surface area (m^2^)	1.9 ± 0.2	1.8 ± 0.2	1.8 ± 0.2^a,b^	0.002
Obesity (%)	8 (24)	30 (36)	50 (22)	0.049
Hypertension (%)	0 (0)	35 (42)	167 (75)	< 0.001
Diabetes (%)	0 (0)	22 (27)	149 (67)	< 0.001
Stroke (%)	0 (0)	0(0)	27 (12)	< 0.001
Vascular disease (peripheral vascular disease, prior myocardial infarction, aortic plaque) (%)	0 (0)	4 (5)	26 (12)	0.028
History of myocardial infarction (%)	0 (0)	4 (5)	26 (12)	0.028
Cigarette smoking (%)	16 (47)	34 (41)	72 (32)	0.133
History of aspirin use (%)	33 (97)	76 (92)	208 (93)	0.561
History of ACEI/ARB use (%)	12 (35)	38 (46)	146 (66)	< 0.001
History of beta-blocker use (%)	24 (71)	55 (66)	170 (76)	0.202
History of calcium-channel blocker use (%)	1 (2.9)	8 (10)	39 (18)	0.031
History of nitrate use (%)	28 (82)	55 (66)	145 (65)	0.132
History of statin use (%)	31 (91)	72 (87)	181 (81)	0.226
History of diuretic use (%)	0 (0)	5 (6)	42 (19)	0.001
History of oral antidiabetic use (%)	0 (0)	16 (19)	110 (49)	< 0.001
History of insulin use (%)	0 (0)	5 (6)	49 (22)	< 0.001
Left anterior descending artery (%)	34 (100)	83 (100)	223 (100)	> 0.999
Left circumflex artery (%)	26 (77)	70 (84)	202 (91)	0.038
Right coronary artery (%)	30 (88)	74 (89)	203 (91)	0.808
Single-vessel disease (%)	3 (9)	4 (5)	4 (2)	0.040
Double-vessel disease (%)	6 (18)	14 (17)	33 (15)	0.853
Triple-vessel disease (%)	25 (74)	65 (78)	186 (83)	0.293
Heart rate (bpm)	64 ± 10	67 ± 11	70 ± 12^a^	0.004
Systolic blood pressure (mm Hg)	124 ± 12	125 ± 15	129 ± 18	0.083
Diastolic blood pressure (mm Hg)	78 ± 8	78 ± 10	76 ± 11	0.356
**Laboratory data**				
Fasting blood sugar (mg/dL)	97 ± 17	113 ± 41^c^	123 ± 42^a^	0.001
Creatinine (mg/dL)	1.1 ± 0.2	1.1 ± 0.2	1.1 ± 0.2	0.620
Hemoglobin (g/dL)	14.8 ± 1.1	14.2 ± 1.7	13.9 ± 1.6^a^	0.009
Triglyceride (mg/dL)	153 (103–200)	145 (117–192)	142 (108–195)	0.790
Cholesterol (mg/dL)	163 ± 50	149 ± 41	141 ± 39^a^	0.009
High-density lipoprotein (mg/dL)	37 (30–41)	35 (30–39)	36 (31–41)	0.641
Low-density lipoprotein (mg/dL)	95 (71–126)	81 (66–109)	77 (62–99)^a^	0.012

ACEI/ARB, Angiotensin-converting enzyme inhibitor/angiotensin II-receptor blocker

^a^Low-risk score group versus high-risk score group

^b^Moderate-risk score group versus high-risk score group

^c^Low-risk score group versus moderate-risk score group

The comparison between the low- and high-risk score groups revealed differences apropos of body surface area and the levels of fasting blood sugar, hemoglobin, cholesterol, and low-density lipoprotein. The level of fasting blood sugar was also different between the low- and moderate-risk score groups. Additionally, a difference was detected vis-à-vis body surface area between the moderate- and high-risk score groups.

### Standard echocardiography data

Standard echocardiography data are presented in Table 2. Differences were detected between the low- and high-risk score groups regarding heart rate at echocardiography time, LV ejection fraction, and LV mass index. In regard to Doppler-based measurements, the 2 groups of low- and high-risk scores exhibited differences in A, the E/A ratio, D, and the S/D ratio. With respect to tissue Doppler imaging-derived indices, the low-risk score group was different from the high-risk score group concerning s′, e′, the e′/a′ ratio, and the E/e′ ratio. As regards LA volumetric parameters, the differences noted between the low- and high-risk score groups were in terms of LAVMx index, LAVMi index, LAVp index, LA total emptying fraction, and LA expansion index.

**Table 2:** Standard echocardiography data of the 3 groups of low-, moderate-, and high-risk CHA_2_DS_2_-VASc scores

Variable	Group			*P* value
	Low-Risk Score (N = 34)	Moderate-Risk Score (N = 83)	High-Risk Score (N = 223)	
LVEDV index (mL/m^2^)	49 ± 8	48 ± 11	53 ± 16	0.014
LVESV index (mL/m^2^)	26 (21–29)	24 (21–29)	28 (21–36)^b^	0.007
LVEF (%)	48 ± 6	47 ± 7	44 ± 9^a,b^	< 0.001
LV mass index (g/m^2^)	77.5 ± 15.2	75.7 ± 17.4	85.7 ± 21.9^a,b^	< 0.001
E (cm/s)	60 ± 13	61 ± 16	63 ± 17	0.327
A (cm/s)	58 ± 14	67 ± 16^c^	80 ± 20^a,b^	< 0.001
E/A ratio	1.1 ± 0.3	0.9 ± 0.3	0.8 ± 0.3^a,b^	< 0.001
DT (ms)	248 ± 33	253 ± 46	241 ± 42	0.088
S (cm/s)	51 ± 8	53 ± 10	53 ± 11	0.435
D (cm/s)	39 ± 8	37 ± 8	35 ± 8^a^	0.034
S/D ratio	1.3 ± 0.2	1.5 ± 0.3^c^	1.5 ± 0.3^a^	< 0.001
Mean s′ (cm/s)	7.8 ± 1.5	7.5 ± 1.4	6.9 ± 1.6^a,b^	< 0.001
Mean e′ (cm/s)	8.2 ± 2.1	7.6 ± 1.7	6.5 ± 1.7^a,b^	< 0.001
Mean a′ (cm/s)	9.1 ± 1.2	9.4 ± 1.4	9.1 ± 1.9	0.608
e′/a′ ratio	0.9 ± 0.3	0.8 ± 0.2	0.7 ± 0.2^a,b^	< 0.001
E/e′ ratio	7.4 (6.3–8.5)	7.9 (6.5–9.7)	9.8 (7.7–12.1)^a,b^	< 0.001
Maximal LA volume index (mL/m^2^)	28 ± 8	30 ± 8	32 ± 8^a,b^	0.003
Minimal LA volume (mL/m^2^)	10 (8–12)	11 (8–14)	12 (9–15)^a^	0.001
Pre-A LA volume (mL/m^2^)	20 ± 6	22 ± 6	24 ± 7^a,b^	0.001
LA total emptying fraction (%)	64 ± 6	62 ± 7	60 ± 9^a^	0.027
Expansion index (%)	188 ± 57	169 ± 50	163 ± 54^a^	0.036
LA passive emptying fraction (%)	28 ± 7	26 ± 7	25 ± 8	0.060
Passive emptying percentage of total emptying (%)	44 ± 9	42 ± 10	41 ± 11	0.264
LA active emptying fraction (%)	50 ± 8	50 ± 7	47 ± 9	0.164
Booster active emptying percentage of total emptying (%)	56 ± 9	58 ± 10	59 ± 11	0.264

^a^Low-risk score group versus high-risk score group

^b^Moderate-risk score group versus high-risk score group

^c^Low-risk score group versus moderate-risk score group

DT, deceleration time; LA, left atrial; LVEDVi, left ventricular end-diastolic volume index; LVEF, left ventricular ejection fraction; LVESVi, left ventricular end-systolic volume index

The low-risk score group was different from the moderate-risk score group regarding A and the S/D ratio. Differences were also detected between the 2 groups of moderate- and high-risk scores in connection with LV end-systolic volume index and LV ejection fraction. With regard to Doppler-based measurements, A and the E/A ratio were also different between the moderate- and high-risk score groups. Apropos tissue Doppler imaging-derived indices, the results demonstrated differences between the 2 groups of moderate- and high-risk scores in terms of s′, e′, the e′/a′ ratio, and the E/e′ ratio. The findings in relation to LA volumetric markers showed that LAVMx index and LAVp index were different between the moderate- and high-risk score groups.

### Two-dimensional Speckle-tracking echocardiography data

**Fig. 2:**
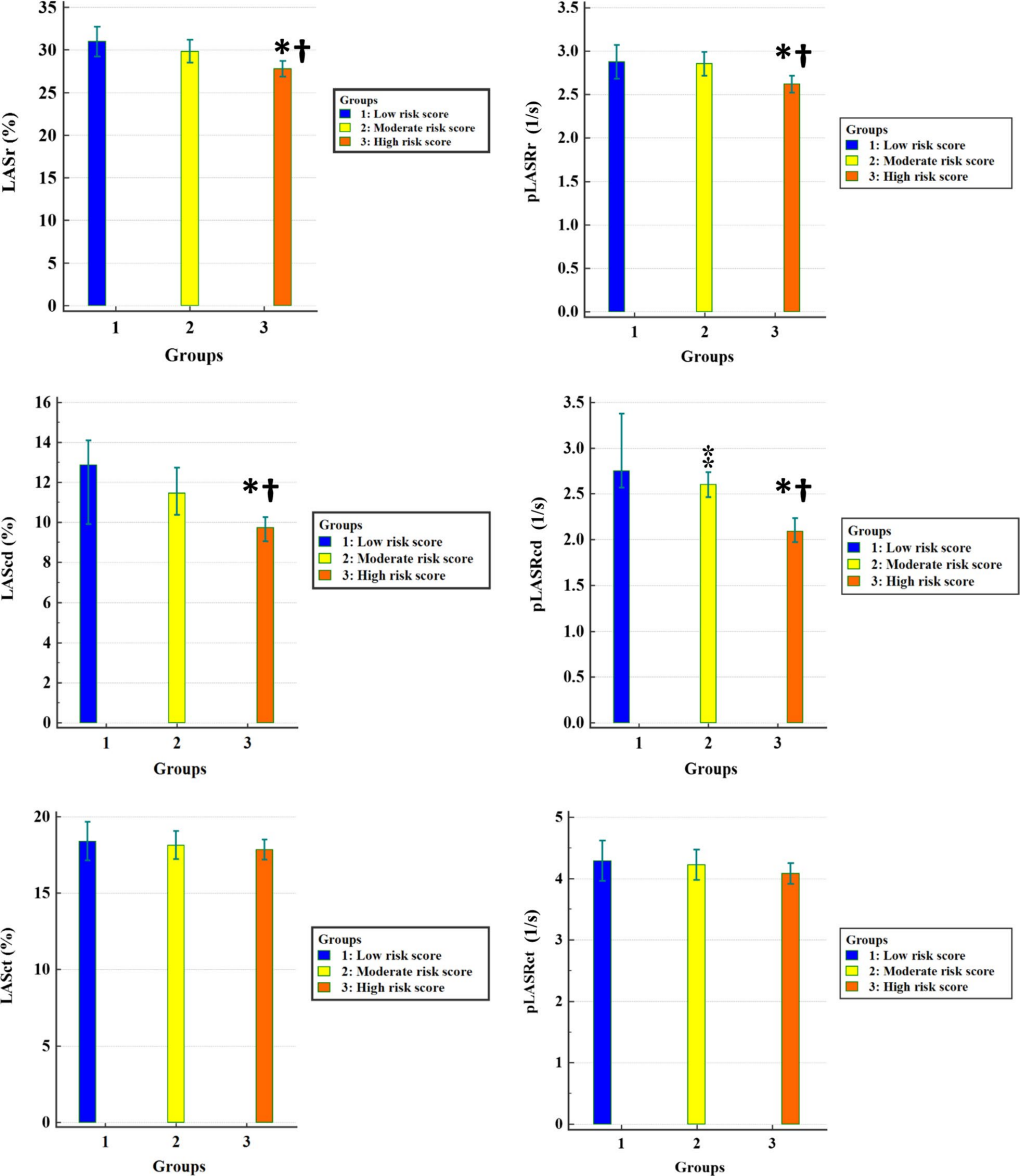
Comparisons are depicted between the low-risk CHA_2_DS_2_-VASc score group and the other 2 study groups. Absolute values are presented. *Low-risk score group versus high-risk score group (*P* < 0.05). ^†^Moderate-risk score group versus high-risk score group (*P* < 0.05). ^⁑^Low-risk score group versus moderate-risk score group (*P* < 0.05). LAScd, Left atrial longitudinal strain during the conduit phase; LASct, Left atrial longitudinal strain during the contraction phase; LASr, Left atrial longitudinal strain during the reservoir phase; pLASRcd, Peak left atrial longitudinal strain rate during the conduit phase; pLASRct, Peak left atrial longitudinal strain rate during the contraction phase; pLASRr, Peak left atrial longitudinal strain rate during the reservoir phase

The 2DSTE-derived indices of LA longitudinal myocardial deformation are presented in Table 3 and Fig. 2. LASr and LASRr (markers of LA reservoir function) and LAScd and LASRcd (markers of LA conduit function) were reduced in the high-risk score group compared with the low- and moderate-risk score groups. No difference constituting statistical significance was found in the comparison between the high-risk score group and the 2 non–high-risk score groups concerning LASct and LASRct (markers of LA contraction function). LASRcd (marker of LA conduit function) was diminished in the moderate-risk score group compared with the low-risk score group. Further, the differences between the low- and moderate-risk score groups in relation to the other longitudinal deformation indices were not statistically significant.

**Table 3:** Comparisons of 2D speckle-tracking echocardiography-derived parameters between 3 groups of low-, moderate-, and high-risk CHA_2_DS_2_-VASc scores

Variable	Group			Unadjusted *P* value	Comparison	Unadjusted post hoc		Adjusted post hoc^b^	
						B^a^ (95% CI)	*P* value	B^a^ (95% CI)	*P* value
	Low-Risk Score Group (N = 34)	Moderate-Risk Score Group (N = 83)	High-Risk Score Group (N = 223)						
LASr (%)	31.0 ± 5.0	29.8 ± 6.1	27.8 ± 6.9	0.004	Low-risk versus moderate-risk scores	1.1 (− 3.7 to 1.5)	0.389	1.4 (− 4.0 to 1.3)	0.313
					Low-risk versus high-risk scores	3.2 (− 5.5 to − 0.8)	0.008	3.7 (− 6.1 to − 1.2)	0.004
					Moderate-risk versus high-risk scores	2.1 (− 3.7 to − 0.5)	0.012	2.3 (− 4.0 to − 0.6)	0.007
pLASRr (1/s)	2.9 ± 0.6	2.9 ± 0.6	2.6 ± 0.7	0.009	Low-risk versus moderate-risk scores	0.0 (− 0.3 to 0.3)	0.881	0.0 (− 0.3 to 0.2)	0.775
					Low-risk versus high-risk scores	0.3 (− 0.5 to 0.0)	0.044	0.4 (− .06 to − 0.1)	0.005
					Moderate-risk versus high-risk scores	0.2 (− 0.4 to − 0.1)	0.007	0.3 (− 0.5 to − 0.1)	< 0.001
LAScd (%)	12.9 (9.4–15.1)	11.5 (9.1–13.8)	9.7 (7.1–12.5)	< 0.001	Low-risk versus moderate-risk scores	0.9 (− 2.5 to 0.8)	0.297	1.1 (− 2.7 to 0.5)	0.191
					Low-risk versus high-risk scores	2.6 (− 4.1 to − 1.1)	0.001	2.7 (− 4.2 to − 1.1)	0.001
					Moderate-risk versus high-risk scores	1.7 (− 2.8 to − 0.7)	0.001	1.6 (− 2.6 to − 0.6)	0.003
pLASRcd (1/s)	2.8 (2.4–3.6)	2.6 (2.2–3.0)	2.1 (1.6–2.7)	< 0.001	Low-risk versus moderate-risk scores	0.4 (− 0.7 to − 0.1)	0.010	0.4 (− 0.8 to − 0.1)	0.005
					Low-risk versus high-risk scores	0.8 (− 1.1 to − 0.5)	< 0.001	0.8 (− 1.1 to − 0.5)	< 0.001
					Moderate-risk versus high-risk scores	0.4 (− 0.6 to − 0.2)	< 0.001	0.4 (− 0.6 to − 0.2)	< 0.001
LASct (%)	18.4 ± 3.6	18.1 ± 4.2	17.8 ± 4.9	0.752	Low-risk versus moderate-risk scores	0.3 (− 2.1 to 1.6)	0.780	0.3 (− 2.1 to 1.5)	0.770
					Low-risk versus high-risk scores	0.6 (− 2.2 to 1.1)	0.513	1.0 (− 2.7 to 0.7)	0.256
					Moderate-risk versus high-risk scores	0.4 (− 1.5 to 0.8)	0.543	0.7 (− 1.9 to 0.4)	0.225
pLASRct (1/s)	4.3 ± 0.9	4.2 ± 1.1	4.1 ± 1.3	0.499	Low-risk versus moderate-risk scores	0.1 (− 0.6 to 0.40)	0.794	0.1 (− 0.6 to 0.4)	0.673
					Low-risk versus high-risk scores	0.2 (− 0.6 to 0.2)	0.359	0.4 (− 0.8 to 0.1)	0.087
					Moderate-risk versus high-risk scores	0.2 (− 0.5 to 0.2)	0.322	0.3 (− 0.6 to 0.0)	0.063

^a^Absolute value of B is presented

^b^Adjusted according to systolic blood pressure, heart rate, hemoglobin levels, low-density lipoprotein levels, cigarette smoking, left circumflex artery stenosis, single-vessel disease, obesity, and nitrate use

CI, confidence interval; LAScd, left atrial longitudinal strain during the conduit phase; LASct, Left atrial longitudinal strain during the contraction phase; LASr, Left atrial longitudinal strain during the reservoir phase; pLASRcd, peak left atrial longitudinal strain rate during the conduit phase; pLASRct, Peak left atrial longitudinal strain rate during the contraction phase; pLASRr, peak left atrial longitudinal strain rate during the reservoir phase

The abovementioned differences remained statistically significant even after adjustments for systolic blood pressure, heart rate, hemoglobin levels, low-density lipoprotein levels, cigarette smoking, left circumflex artery stenosis, single-vessel disease, obesity, and nitrate use. The results of the intra- and interobserver variabilities are presented in Table 4.

**Table 4:** Intra- and interobserver variabilities for the 2D speckle-tracking echocardiography-derived indices of left atrial myocardial function

Variable	Intraobserver		Interobserver	
	ICC	95% limit of agreement	ICC	95% limit of agreement
LASr (%)	0.994	0.988–0.997	0.949	0.907–0.972
LAScd (%)	0.994	0.989–0.996	0.931	0.684–0.974
LASct (%)	0.992	0.984–0.996	0.921	0.849–0.958
pLASRr (1/s)	0.992	0.981–0.996	0.942	0.850–0.973
pLASRcd (1/s)	0.996	0.993–0.998	0.973	0.952–0.985
pLASRct (1/s)	0.993	0.977–0.997	0.944	0.897–0.969

ICC, intraclass correlation coefficient; LAScd, left atrial longitudinal strain during the conduit phase; LASct, left atrial longitudinal strain during the contraction phase; LASr, left atrial longitudinal strain during the reservoir phase; pLASRcd, peak left atrial longitudinal strain rate during the conduit phase; pLASRct, peak left atrial longitudinal strain rate during the contraction phase; pLASRr, peak left atrial longitudinal strain rate during the reservoir phase

### Clinical outcomes

Clinical outcome data are presented in Table 5. The risk of in-hospital mortality and prolonged mechanical ventilation in the high-risk score group was not statistically significantly different from that of the 2 non–high-risk score groups (*P* > 0.05). The risk of postoperative AF and prolonged ICU stay was statistically significantly more in the high-risk score group than the 2 non–high-risk score groups [9.67 (1.31–71.43), *P* = 0.026 and 8.05 (1.08–60.16), *P* = 0.042, respectively].

**Table 5:** Clinical outcome comparisons between 2 groups of non–high-risk and high-risk CHA_2_DS_2_-VASc scores

Group	Variables			*P* value
	Non-High-Risk^a^ Score (N = 109)	High-Risk Score (N = 203)	RR, 95% CI	
Mortality	2 (2)	6 (3)	1.61 (0.33–7.85)	0.555
Postoperative atrial fibrillation	1 (1)	18 (9)	9.67 (1.31–71.43)	0.026
Prolonged ICU stay	1 (1)	15 (7)	8.05 (1.08–60.16)	0.042
Prolonged mechanical ventilation	2 (2)	11 (5)	2.95 (0.67–13.09)	0.154

^a^Non-high-risk group was comprised of 30 patients with low risk scores and 79 patients with moderate risk scores

CI, confidence interval; ICU, intensive care unit; RR, relative risk

## Discussion

In the present study, we assessed the ability of the CHA_2_DS_2_-VASc scoring system to identify LA dysfunction in patients with sinus rhythm who were candidates for CABG. We found that LA conduit function was decreased in patients with moderate- and high-risk scores by comparison with those with mild-risk scores and also in patients with high-risk scores in comparison with those with moderate-risk scores. LA reservoir function was also lower in patients with high-risk scores than in those with mild- and moderate-risk scores. In addition, the risk of postoperative AF and prolonged ICU stay was highest in the high-risk score patients, who also had the worst LA reservoir and conduit functions of the 3 study groups.

To the best of our knowledge, we are the first to assess the efficacy of the CHA_2_DS_2_-VASc scoring system in categorizing all 3 LA functions as evaluated by longitudinal strain and strain rate markers in patients with sinus rhythm. Previous investigations have evaluated the ability of the CHADS_2_ and CHA_2_DS_2_-VASc scoring systems to categorize LA function in patients with AF by focusing solely on LASr [21–23]. We categorized our study population via the CHA_2_DS_2_-VASc scoring system in compliance with the latest recommendations of the American College of Cardiology/American Heart Association, which consider the factor of sex to be an effect modifier [24].

Saha et al. categorized their patients with AF with the aid of the CHADS_2_ scoring system as mild-, moderate-, and high-risk score groups and reported a drop in LASr in the entire study population irrespective of the categorization [21]. Saha and colleagues, however, failed to perform pairwise comparisons between their 3 groups and regarded CHADS_2_ scores of 2, 2 and 3, and greater than 3 as mild-, moderate-, and high-risk scores, respectively, which is in stark contrast to what we did in our study. In their investigation on patients with AF, Li et al. reported diminished LASr in their moderate- and high-risk score groups compared with their mild-risk score group [22]. Moreover, they found no significant difference between the moderate- and high-risk score groups concerning LASr. Li and coworkers considered CHA_2_DS_2_-VASc scores of 0, 1, and 2 or greater to represent mild-, moderate-, and high-risk scores, which is different from our categorization. Islas et al. by using 3D wall-motion tracking demonstrated a decrease in LA longitudinal systolic strain in tandem with an increase in CHA_2_DS_2_-VASc scores in patients with AF [23]. Nevertheless, they failed to perform pairwise comparisons between their study groups and considered CHA_2_DS_2_-VASc scores of 0 and 1, 2 and 3, and greater than 3 to represent mild-, moderate-, and high-risk scores, which does not chime in with our study.

Previous studies have demonstrated that the factors that constitute the CHA_2_DS_2_-VASc scoring system such as congestive heart failure [30], LV dysfunction [30], hypertension [31], age [32], diabetes [33], stroke [34], sex (female) [35], and prior myocardial infarction [36] can all individually have an impact on LA function. Accordingly, an increase in the risk score of these factors denotes further impairment in LA function.

Be that as it may, the CHA_2_DS_2_-VASc scoring system suffers from certain inherent weaknesses. Firstly, it simply allocates similar scores to the effects of different factors on LA function. By way of example, it considers the same risk score for the impact of hypertension, diabetes, and prior myocardial infarction on LA function, which cannot be representative of the actual severity of the effect of each on the different functions of LA. Secondly, the system was originally developed for the prediction of a clinical event, but not the categorization of functional impairment. Notwithstanding these shortcomings, the results of our study showed that not only was the CHA_2_DS_2_-VASc scoring system able to identify reduced LA reservoir and conduit functions in patients with high-risk scores (CHA_2_DS_2_-VASc scores ≥ 2 for men and ≥ 3 for women) but also it was capable of identifying reduced LA conduit function in patients with moderate-risk scores compared with those with low- and high-risk scores.

LA function is not completely independent of LV systolic and diastolic functions insofar as LV systolic and diastolic dysfunction can lead to LA dysfunction [37, 38]. The factors incorporated in the CHA_2_DS_2_-VASc scoring system can impair LV systolic and diastolic functions. In systole, impaired LV contraction decreases the displacement of the mitral annulus and diminishes the stretching of LA myocardial fiber. Ejection fraction and s′, as markers of LV systolic function, were reduced in our high-risk score group compared with our low- and moderate-risk score groups, which justifies the reduction in LA reservoir function. In diastole, impaired LV relaxation lowers the speed of the movement of the mitral annulus toward its reference level and concurrently, exposes the LA to an elevation in its filling pressure, preventing the exit of blood from the LA to the LV. If we consider e′ to be a marker of LV diastolic function and the E/e′ ratio to be an index of LV filling pressure, the diminished LA conduit function in our high-risk score group is expectable. In addition, the diastolic function of the LV is more sensitive to damage than its systolic function, which may explain the difference between the moderate-risk score group and the other 2 groups concerning LA conduit function in the current study. Still, apropos LA reservoir function, we found no difference between our moderate- and low-risk score groups. What should also be taken into account is the evidence suggesting that factors such as diabetes and hypertension can independently damage LA function and beget LA dysfunction earlier than LV dysfunction [39, 40]. Such evidence is bolstered by the difference between the time-to-peak systolic strain of the LV and LASr [41].

The factors incorporated in the CHA_2_DS_2_-VASc scoring system damage LA function through several mechanisms such as fibrosis, insulin resistance, oxidative stress in patients with diabetes [42], fibrosis in aging [43], hypertension [44], estrogen effect in postmenopausal women [45], neurohormonal activation in heart failure [46], renin–angiotensin–aldosterone system activation in patients with myocardial infarction [47], and occult AF in patients with stroke [48].

The volumetric parameters of LA reservoir function were different between our 3 study groups; this is further evidence in support of our findings via 2DSTE, although this echocardiography modality evaluates LA function indirectly and is subject to geometrical assumptions.

Our results revealed the highest risk of postoperative AF and prolonged ICU stay among the high-risk score patients, who also had worse LA reservoir and conduit functions than the 2 non–high-risk score groups. The increased risk of postoperative AF after CABG in concurrence with increased CHA_2_DS_2_-VASc scores have been previously reported [9–11], which is in line with our small-scale study. The prolonged ICU stay after cardiac surgeries such as CABG is correlated with elevated LV diastolic filling pressure as evaluated by the E/e′ ratio, which a marker of LV filling pressure, and LA function, which is correlated with LV diastolic function [49]. The detrimental effect of LV diastolic dysfunction on LA function [50] may explain our finding, especially given the rise in the E/e′ ratio in our high-risk score group.

From a clinical perspective, our study results imply that the CHA_2_DS_2_-VASc scoring system is an easy and rapid method that relies only upon clinical data and, thus, may be interchangeable with the implications of diminished LA reservoir and conduit functions. The clinical importance of this scoring system for the classification of LA function is better delineated by the existing evidence indicating that the evaluation of the function of this chamber can discriminate between heart failure with preserved ejection fraction and noncardiac causes of dyspnea [51]. Assessments of LA function confer information that is more accurate than that obtained based on the current guidelines for the classification of LV filling pressure [52]. Moreover, LA function is capable of predicting adverse cardiovascular events both in patients suffering from heart failure with reduced or preserved ejection fraction [53, 54] after myocardial infarction [55] and in the general population [56]. A recent study demonstrated that a reduction in the reservoir, conduit, and contraction functions of the LA before CABG was able to predict the incidence of heart failure and/or cardiovascular death up to 7 years after surgery [57].

Although it may seem that our exclusion criteria limited the generalizability of our results, our exclusion criteria contained conditions that probably required additional surgical procedures or which were capable of skewing the measurement of LA longitudinal deformation markers because of their impact on LA function were not included in the CHA2DS2-VASc scoring system. In sum, these factors could confound echocardiography measurements and clinical events.

### Study limitations

The study population of the present cross-sectional, single-center study was limited to CABG candidates, limiting the generalizability of the results to other patient groups. The low sample size, especially in the low- and moderate-risk score groups, limited the power of our study to detect not only differences regarding the markers of LA contraction function between all 3 study groups but also differences concerning LA reservoir function indices between the low- and moderate-risk score groups. Our results would have been more robust had we been able to use 3D echocardiography, cardiac magnetic resonance imaging, invasive measurements of LV filling pressure, or electrocardiography Holter monitoring. Another drawback of our study was the exclusion of patients with a history of any type of symptomatic AF, which means that some of our patients may have had asymptomatic paroxysmal AF. That we failed to follow our patients as regards the occurrence of clinical events after hospital discharge and we used software primarily designed for the evaluation of the LV can also be deemed weaknesses.

## Conclusions

The results of our study demonstrated that the CHA_2_DS_2_-VASc scoring system was able to categorize the patient population not only in regard to LA conduit function as low-, moderate-, and high-risk score groups inasmuch as this function decreased throughout this continuum but also with respect to LA reservoir function as high-risk and non–high-risk score groups insofar as this function decreased throughout this spectrum. These findings were further supported by the finding of an increased risk of postoperative AF and prolonged ICU stay in the high-risk group by comparison with the non–high-risk score groups.

## Data Availability

The data sets analyzed in the current study are available from the corresponding author on reasonable request.
